# Effect of Short-Term Exposure to Fine Particulate Matter and Temperature on Acute Myocardial Infarction in Korea

**DOI:** 10.3390/ijerph18094822

**Published:** 2021-04-30

**Authors:** Jiyoung Shin, Jongmin Oh, In-Sook Kang, Eunhee Ha, Wook-Bum Pyun

**Affiliations:** 1 Department of Occupational and Environmental Medicine, College of Medicine, Ewha Womans University, Seoul 07804, Korea; nellshin5@gmail.com (J.S.); jongminoh@ewha.ac.kr (J.O.); 2 Inflammation-Cancer Microenvironment Research Center, College of Medicine, Ewha Womans University, Seoul 07804, Korea; 3 Department of Internal Medicine, Division of Cardiology, College of Medicine, Ewha Womans University, Seoul 07804, Korea; pinkvision21@gmail.com; 4 Graduate Program in System Health Science and Engineering, College of Medicine, Ewha Womans University, Seoul 07804, Korea

**Keywords:** fine particulate matter, temperature, acute myocardial infarction, national health information database, time-series study

## Abstract

Background/Aim: Previous studies have suggested that the short-term ambient air pollution and temperature are associated with myocardial infarction. In this study, we aimed to conduct a time-series analysis to assess the impact of fine particulate matter (PM2.5) and temperature on acute myocardial infarction (AMI) among adults over 20 years of age in Korea by using the data from the Korean National Health Information Database (KNHID). Methods: The daily data of 192,567 AMI cases in Seoul were collected from the nationwide, population-based KNHID from 2005 to 2014. The monitoring data of ambient PM2.5 from the Seoul Research Institute of Public Health and Environment were also collected. A generalized additive model (GAM) that allowed for a quasi-Poisson distribution was used to analyze the effects of PM2.5 and temperature on the incidence of AMI. Results: The models with PM2.5 lag structures of lag 0 and 2-day averages of lag 0 and 1 (lag 01) showed significant associations with AMI (Relative risk [RR]: 1.011, CI: 1.003–1.020 for lag 0, RR: 1.010, CI: 1.000–1.020 for lag 01) after adjusting the covariates. Stratification analysis conducted in the cold season (October–April) and the warm season (May–September) showed a significant lag 0 effect for AMI cases in the cold season only. Conclusions: In conclusion, acute exposure to PM2.5 was significantly associated with AMI morbidity at lag 0 in Seoul, Korea. This increased risk was also observed at low temperatures.

## 1. Introduction

Acute myocardial infarction (AMI) is a leading cause of death worldwide [1,2]. Similar to other countries, the incidence of AMI in Korea has increased over the last few decades [3,4]. Therefore, it is important to prevent the occurrence of AMI and identify the risk factors of AMI.

Many environmental factors have been suggested as risk factors for AMI. Air pollution has been repeatedly associated with increased risks of hospital admissions and deaths due to cardiovascular disease. Specifically, particulate matter (PM) has been identified as a risk factor for cardiovascular disease in studies performed throughout the industrialized world [5,6,7]. Air pollution is increasing as urbanization and industrialization processes expand worldwide.

Recently, growing evidence has shown that fine particulate matter (PM2.5), which is ≤2.5 µm in aerodynamic diameter, may play a role in the development of cardiovascular diseases and that different sizes of particulate matter affect cardiovascular health differently [8,9,10]. Previous research suggested that although smoking is a more important risk factor for cardiovascular mortality, exposure to PM2.5 is also a risk factor for the disease via mechanisms including oxidative stress and systemic inflammation [5,11]. Some studies have also shown that both short- and long-term particulate matter exposure are specific triggers of myocardial infarction (MI) [5,6]. A multicity study in China examined the short-term effects of air pollution on AMI morbidity and found that air pollutants, including PM10, were positively associated with the daily AMI admissions at lag 2 and lag 3 days [12].

In addition to the PM2.5, the temperature can also be a risk factor for adverse cardiovascular outcomes. Previous studies suggested that high or low temperatures are associated with the AMI mortality and morbidity. For instance, studies conducted in Cuba, Sweden, Massachusetts and Denmark reported an increased AMI risk at low temperatures [13,14,15,16]). Other studies conducted in South Korea and England identified that both low and high temperatures are associated with an increased AMI risk [17,18].

South Korea has serious air pollution concerns due to the frequent haze events and PM2.5 concentrations in recent years [19,20]. Seoul is the largest city in South Korea and it is a highly urbanized area with many emission sources, such as automobile exhaust, industrial factories, and power-generating facilities [21]. Furthermore, because of local and regional emission, and meteorological and chemical interactions, there is a high concentration of PM2.5 in Seoul.

The temperature of South Korea is also unique when it is compared to the other countries. This is because South Korea has four distinct seasons and a relative wide temporal variation in climate. The weather of South Korea is different between the central and southern parts of the Korean peninsula, and the central part, including Seoul, is colder than the southern parts [17].

Therefore, it is very important to identify the effect of PM2.5 and temperature on acute myocardial infarction (AMI) events in Seoul, Korea, which is highly polluted by PM2.5. We conducted a population-based study designed to investigate the impact of PM2.5 and temperature on acute myocardial infarction (AMI) among adults over 20 years of age in Seoul, Korea, by using data from the Korean National Health Information Database (KNHID).

## 2. Methods

### 2.1. Data Source

This study used data from the Korean National Health Information Database (KNHID) collected between 1 January 2005 and 31 December 2014. The KNHID contains information about participants who visited hospitals under the Korean National Health Insurance Service (NHIS) program [22]. Since this National Health Insurance Service in Korea is a single-payer program and is mandatory for all residents, the KNHIS represents the entire Korean population and can be utilized as a population-based database [23]. The KNHID includes five databases (an eligibility database, a national health screening database, a healthcare utilization database, a long-term care insurance database, and a medical institution database) and contains data on the diagnosis and status of outpatients and inpatients [23].

The monitoring data of ambient PM2.5 from the Seoul Research Institute of Public Health and Environment were also collected. In Korea, the city of Seoul has recognized the importance of exposure to PM2.5 and began measuring PM2.5 in early 2000. Daily PM2.5 values during the study period between 1 January 2005 and 31 December 2014, were taken from 25 monitoring sites installed in the administrative districts of Seoul using the SPM-613D beta gauge method [24]. The daily PM2.5 values in Seoul were computed by averaging the daily mean concentration of PM2.5 at all monitoring stations.

To estimate other co-pollutants, including CO, SO_2_, and NO_2_, we obtained complete air pollution data from local district air quality fixed-site monitoring stations in Korea managed by the National Ambient Air Monitoring System [25]. Because the data on air pollution were not available at all administrative sites in Seoul, we applied kriging models to derive exposure assessments using geographic information systems (GIS) tools (ArcGIS Version 9.3, ESRI, Redlands, CA, USA) to estimate ambient air pollution levels in unmonitored districts. In addition, kriging interpolation considered a semivariogram model, and to evaluate the performance of the kriging model, the root mean square standardized error was referred to. Daily values were computed by averaging the daily mean concentration of air pollutants at all administrative districts in Seoul.

Weather condition data, including daily average temperature, daily maximum temperature, daily minimum temperature, daily mean relative humidity, and dew-point temperature were acquired from the database of the Korea Meteorological Administration (KMA), which runs 76 automated weather stations in the Seoul metropolitan area.

### 2.2. Ethic Approval

This study was approved by the Institutional Review Board of Ewha Womans University Hospital, Seoul, Republic of Korea (IRB number: EUMC 2019-12-009).

### 2.3. AMI Events and Exposure Definition

We obtained the study population data from the KNHID between 1 January 2005 to 31 December 2014. The AMI patients were defined as persons who were newly diagnosed under a diagnostic code for AMI. Only the day of the first diagnosis of AMI was designated as a day with an AMI event. We identified the AMI diagnosis from the patient’s medical treatment based on the Korean Classification of Diseases, 6th revision (KCD-6), which is a modified version of the International Classification of Disease, 10th revision (ICD-10). We set the criteria of AMI patients based on the KCD-6 code for acute myocardial infarction (codes I21). Data regarding 192,567 AMI events in Seoul were collected during the study period. For the temperature exposure, we used three daily temperatures including daily mean temperature, daily maximum temperature, and daily minimum temperature.

### 2.4. Statistical Analysis

A time-series design was used to analyze the daily data of AMI cases, PM2.5 concentration and weather variables that were linked by date. Since the variance of daily AMI cases is greater than the mean, the daily AMI cases assumed a quasi-Poisson distribution (Supplementary Table S1). We fit a generalized additive model (GAM) to identify the association between PM2.5 and AMI cases:

(1) $$\begin{array}{c} \mathrm{Log}\left[E\left(Y_{t}\right)\right]=\;\mathrm{intercept}+\beta \;\times {\;\mathrm{PM}}_{2.5}+s\left(\mathrm{Calendar}\;\mathrm{time},\;df=4\times 10\right)+\\ s\left(\mathrm{Temperature},df=6\right)+s\left(\mathrm{Dew}\;\mathrm{point}\;\mathrm{temperature},df=3\right)+s\left(\mathrm{relative}\;\mathrm{humidity},df=5\right)+\mathrm{day}\;\mathrm{of}\;\mathrm{week} \end{array}$$

where $E\left(Y_{t}\right)$ represents the number of AMI events at day *t*; s shows smoothing spline and β represents the log-relative risk of AMI morbidity associated with a unit increase of PM2.5. Relative risks (RR) of AMI morbidity with a 10$\;\mu g/m^{3}$ increase in PM2.5 concentration was calculated. To control for the seasonal patterns and long-term trends, we considered smoothing spline for calendar time with 4 degrees of freedom per year to control for seasonal trends, temperature with 6 degrees of freedom, dew point temperature with 3 degrees of freedom and relative humidity with 5 degrees of freedom [26]. The day of the week was controlled as a categorical variable. We used the value of temperature, dew point temperature, and relative humidity at lag 0 as a covariate. Degrees of freedom (*df*) were further tested by the sensitivity analyses.

To figure out the relationship between ambient PM2.5 exposures and AMI morbidity, we fit the models with different lag structures from lag 0 days (at the day of the AMI diagnosis) to lag 3 days. To consider the exposure effects of the average over the same and previous days, we used the 2-day to 4-day moving averages of PM2.5 to estimate the association between PM2.5 exposure and AMI morbidity [27]. We also explored the effect of three daily temperatures on AMI morbidity in adults.

In the main model, we assumed that the relationship between exposure variables (i.e., PM_2.5_) and AMI events was linear. However, the exposure–response relationship may also indicate non-linearity. Therefore, we visualized the exposure–response relationship between the exposure variables (PM_2.5_ and temperature) and the AMI events.

The stratified analysis of PM_2.5_ concentration and daily AMI events was further carried out in the cold months (October–April) and warm months (May–September) separately [28,29]. We also defined the daily mean temperature strata in four levels (<3.70 °C, 3.70–14.30 °C, 14.30–22.40 °C, and ≥22.40 °C) by using quartiles and examined the relationship within each group.

All *p* values were 2-sided, and those less than 0.05 were considered to be significant. The statistical analyses were performed using the R (version 3.5.2) ‘mgcv’ package (R development Core Team, Vienna, Austria).

## 3. Results

### 3.1. Summary Statistics of Air Pollutant and Weather Conditions

Table 1 shows the summary statistics of air-pollutant concentrations and meteorological indicators including daily temperature, relative humidity and dew point temperature. During the 10 years under study, the mean daily PM2.5 concentration was 25.7$\;\mu g/m^{3}$. The mean daily average temperature was 12.7 °C and the average daily maximum temperature was 17.1 °C. The average relative humidity was 60.6% during the study period. The concentrations of PM2.5 were higher in the cold season. The daily average temperature was 22.8 °C in the warm season and 5.39 °C in the cold season (Supplementary Table S2).

### 3.2. AMI Incidence

Supplementary Table S3 represents the newly diagnosed AMI patients enrolled in the NHID in the overall cities and Seoul in Korea from 2005 to 2014. Of the 192,567 AMI cases that occurred during the study period, 17.27% of the data were cases that occurred in Seoul.

The crude incidence in Seoul was lower than the overall crude incidence. In 2005, the incidence of AMI was the highest in Seoul. In 2005, the crude incidence per 100,000 was 46.4. In 2011, the crude incidence per 100,000 in Seoul was 39.3, which was the lowest incidence during the study period.

### 3.3. Relative Risk Estimates for Cases of AMI Events

RR and 95% CI regarding the relationship between 10 $\mu g/m^{3}$, the increase in PM2.5 at different exposure days and daily cases of AMI are summarized in Table 2. In the single pollutant models, the models with lag structures of lag 0 and lag 01 showed significant associations with AMI (RR: 1.011, CI: 1.003–1.020 for lag 0, RR: 1.010, CI: 1.000–1.020 for lag 01). In the co-pollutant model which simultaneously included two pollutants (PM2.5 and alternatively NO2, SO2 or CO), we could observe significant associations between PM2.5 and AMI events in the lag 0 model only.

**Figure 1:**
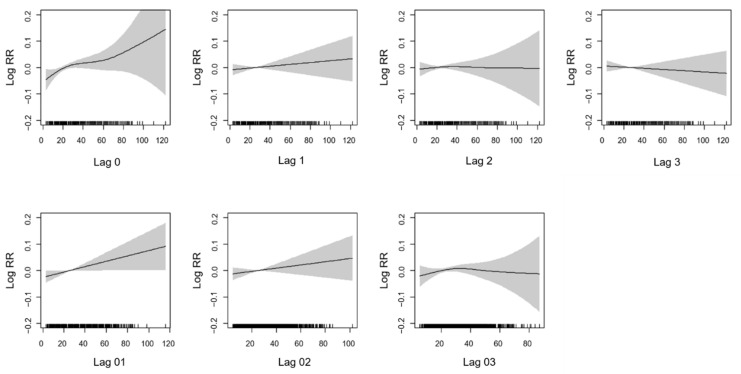
The exposure-response curve for the association between concentration of PM2.5 and AMI over lag days. The black line is the log relative risk, and the gray area is the 95% confidence intervals of the risk estimates.

Figure 1 presents the average exposure–response curve between PM2.5 concentrations and the risk of daily AMI events. There were exposure–response relationships of the PM2.5 concentration and AMI at lag 0, lag 1, and the 2-day averages of lags 0 and 1 (lag 01). We also noted a broadly linear association for the 3-day average of lags 0, 1 and 2 (lag 02). A longer lag association show relatively flat or negative curves at lag3 and lag 03.

**Figure 2:**
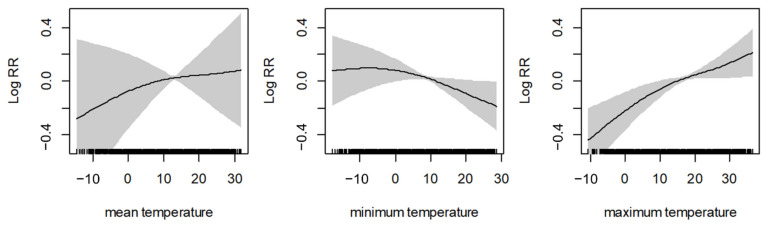
The nonlinear curve for the association between different temperatures and AMI. The black line is the log relative risk, and the gray area is the 95% confidence intervals of the risk estimates.

Figure 2 shows the nonlinear curve for the association between three different temperatures (mean temperature, minimum temperature, and maximum temperature) and AMI. The results show a non-linear positive relationship between mean temperature and AMI. Furthermore, if the maximum temperature level increased, the log relative risk of AMI also increased nonlinearly. However, when we assumed the linear effect of the temperatures and identified the relationship between the three daily temperatures and daily cases of AMI events, the results showed no significant relationships between temperatures and AMI (Supplementary Table S4).

Table 3 shows the adjusted RR for daily cases of AMI events per 10$\;\mu g/m^{3}$ increase in PM2.5 at different lag days, which is stratified by the quartile level of daily mean temperature at lag 0. The results showed when the daily mean temperature level at lag 0 was between 3.70 and 14.30 °C, a 10$\;\mu g/m^{3}$ increase in PM2.5 at lag 0, lag 01 and lag 02 were significantly associated with increased daily cases of AMI events. In the co-pollutant models at Supplementary Table S6, PM2.5 at lag 0 were significantly associated with AMI events, and Lag 01 was only associated with AMI in SO2 adjusted model. In different temperature groups such as <3.70 °C, 14.30–22.40 °C, and ≥22.40, the results did not show any significant associations.

**Figure 3:**
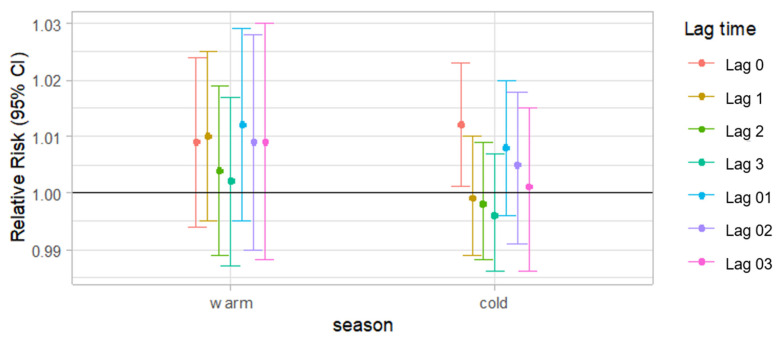
Adjusted relative risk for daily cases of AMI events (95% CI) per 10$\;\mu g/m^{3}$ increase in PM2.5 t single-lag (lag 0, lag 1, lag2 and lag3) and cumulative-lag (lag 01, lag 02 and lag 03) models according to the season ^a^. ^a^ Results adjusted for calendar time, daily mean temperature, dew-point temperature, relative humidity and day of the week.

Figure 3 shows the results of stratified analyses by season. The associations between PM2.5 concentration and the cases of AMI events varied by season. The stratified analysis carried out in the cold season (October–April) and the warm season (May–September) showed a significant lag0 effect for AMI cases only in the cold season (RR: 1.012, CI: 1.002–1.023) after the adjustment for the covariates. Except for the lag0 effect, the other lag effects were not significant.

## 4. Discussion

This study suggests there is evidence of an association between PM2.5 concentration and AMI morbidity in adults at lag 0 and lag 01 in Seoul, Korea. However, the adverse effect of PM2.5 at lag01 became insignificant after adjustment for other co-pollutants. Our results are mostly consistent with previous studies that reported detrimental heart effects from short-term exposure to PM2.5. A cohort study performed in Beijing showed a significant association between PM2.5 concentration and ischemic heart disease (IHD) morbidity in lag 0 and lag 0–2 days [30], which is consistent with our study. Furthermore, a study conducted in Belgium, which has a lower level of particulate matter than Seoul, demonstrated a significant effect of PM10 on AMI events at lag day3. However, the unconstrained distributed lag model did not show a significant relationship [31].

In the stratified analyses by the season and daily mean temperature, the relationship was significant only in the cold season. Furthermore, when the daily mean temperature at lag 0 was between 3.70 and 14.30 °C, the increase of PM2.5 at lag 0, lag 01 showed significant positive associations with the daily cases of AMI events. In March and April, the mean daily temperature is 5.47 and 12.0 °C, and Asian dust from dust storms is common in Korea. Therefore, this seasonal phenomenon can be another factor contributing to cardiovascular events [32]. In the warm season, the PM2.5 concentration was not associated with the AMI morbidity. One possible reason for the significant association in the cold season is connected to a low temperature and increased blood pressure and viscosity in the cold season, which might be important causal factors in increasing winter morbidity due to heart attacks and strokes [33,34].

Our results indicate the effect estimates were higher in the cold season, but they did not show that if the temperature drops the risk increases since the daily mean temperature group <3.70 °C showed a negative relationship between PM2.5 increase and the risk of AMI. In very cold weather, people stay home or inside. This activity pattern might attenuate the effect of the lowest temperature group on AMI, and thus only the medium temperature group shows a significant positive association between PM2.5 and AMI events. A study of seasonal variations in hospital admissions with AMI in Korea also found that AMI events increased during October to December (daily mean temperature in October to December: −1.00 to 15.5 °C) and then reduced in January to February (daily mean temperature of January and February: −2.46 and 0.50 °C) [35].

Analyses about effect modification by season or temperature was performed in other studies conducted in other countries and some results were consistent with our results. A previous time-series study conducted in Shanghai, China reported that the daily counts of coronary heart disease (CHD) morbidity in the cold season (November–April) were higher than those in the warm season (May–October) [36]. They also found a significant association between the particulate matter concentration, including PM2.5 and PM10 at lag 01 and CHD outpatient and emergency department visits in all seasons. Although these significant particulate matter effects were observed in all seasons and cold seasons, the association was not statistically significant in the warm season. Research conducted in Hong Kong also found detrimental effects of air pollution in cool and dry seasons. In cool and dry seasons, a 10$\;\mu g/m^{3}$ increment of lag 03 exposure was associated with an increase in emergency IHD admissions. However, another study conducted in Belgium showed a steep linear association in summer, whereas in winter, the association was non-linear [37]. Studies conducted in U.S. cities also showed that for the 10 µg/m^3^ increase in 2-day averaged PM2.5, the percent increases in all mortality categories were greatest in the spring [38].

For the strengths of our study, although many studies have analyzed the association between PM2.5 concentration and heart diseases, such as in the UK, US and other developed countries, those PM2.5 exposure levels are quite low [39,40]. Therefore, it is important to identify the relationship between PM2.5 exposure and heart disease in a highly polluted region. Our average PM2.5 level during the study period was 25.7$\;\mu g/m^{3}$, which is higher than the World Health Organizations’ Air Quality Guidelines (10$\;\mu g/m^{3}$, annual mean), USA National Ambient Air Quality Standards (12$\;\mu g/m^{3}$, annual mean) and Korean air-quality standards (15$\;\mu g/m^{3}$, annual mean) [25,41,42]. We conducted the study in Seoul, a moderately polluted area with PM2.5, and found effects at higher levels of exposure. Another strength of our study is that we tried to avoid overestimating the effect of PM2.5 by constructing the co-pollutant models, and therefore we constructed both single pollutant and co-pollutant models to consider the potential role of other gaseous air pollutants such as CO, SO_2_, and NO_2_. Finally, our study is a large-scale, population-based study used KNHID data. We also used KCD codes to identify the AMI patients, which increased AMI diagnosis accuracy as it was confirmed by a physician.

For the limitations of this study, since our PM2.5 data from the Seoul Research Institute of Public Health and Environment were available only in Seoul, from 1 January 2005, we could not extend the analysis to before 2005, or to other locations except Seoul. Secondly, the air pollution level of the day was estimated according to the average of all monitoring sites in Seoul, Korea. Therefore, the possible diversity of the exposure level of each patient such as in the working area, indoor or outdoor daily activity, or the distance from the monitoring station to the patients’ homes were not considered in the exposure estimation.

In conclusion, our results have demonstrated that the PM2.5 concentration was significantly associated with an increased AMI morbidity at lag 0 in Seoul, Korea. This increased association was also observed at low temperatures, suggesting that the temperature could modify the effect of air pollution on cardiovascular outcomes. Further studies from other countries that have different temperature trends are needed to examine the effect modification of PM and AMI association by temperatures. Moreover, considering the increasingly aging population, future studies identifying the effect of air pollution and temperature exposure on cardiovascular events in elderly adults are needed.

## Figures and Tables

**Table 1:** Average air-pollutant concentration and weather conditions during the study period (2005 to 2014).

Air Pollutants	Mean ± SD	Min	P25	Median	P75	Max
PM2.5 ^a^ ($\mu g/m^{3}$)	25.7 ± 14.2	3.0	16.0	23.0	32.0	122
CO (ppb)	590 ± 241	218	427	529	683	1835
SO2 (ppb)	5.46 ± 2.26	2.30	3.86	4.87	6.45	22.0
NO2 (ppb)	33.98 ± 12.37	6.50	24.4	32.2	42.2	89.4
Meteorological indicators						
Daily average temperature (°C)	12.7 ± 10.6	−14.5	3.7	14.3	22.4	31.8
Daily maximum temperature (°C)	17.1 ± 10.8	−10.7	7.8	19.0	26.6	36.7
Daily minimum temperature (°C)	8.96 ± 10.7	−17.8	−0.2	9.9	18.7	28.7
Relative humidity (%)	60.6 ± 15.0	19.9	49.4	60.6	71.5	99.8
Dew point temperature (°C)	4.55 ± 12.1	−25.4	−5.20	5.15	15.1	25.3

^a^ PM2.5: particulate matter <2.5 µm in aerodynamic diameter; SD: standard deviation; Min: minimum; Max: maximum, P25: 25th percentile, P75: 75th percentile.

**Table 2:** Adjusted relative risk estimates for daily cases of AMI event (95% CI) per 10 µg/m^3^ increase in PM2.5 at single-lag (lag 0, lag 1, lag 2 and lag 3) and cumulative-lag (lag 01, lag 02 and lag 03) models ^a^.

Lag Day	RR (95% CI)			
	None	+CO	+SO2	+NO2
Lag 0	1.011 (1.003–1.020) *	1.018 (1.005–1.031) *	1.013 (1.000–1.025) *	1.013 (1.001–1.024) *
Lag 1	1.004 (0.995–1.012)	0.998 (0.985–1.011)	1.005 (0.992–1.017)	0.993 (0.982–1.004)
Lag 2	1.000 (0.991–1.009)	0.999 (0.986–1.012)	1.000 (0.988–1.013)	0.995 (0.984–1.006)
Lag 3	0.997 (0.989–1.006)	0.999 (0.986–1.012)	1.003 (0.991–1.015)	0.996 (0.985–1.007)
Lag 01	1.010 (1.000–1.020) *	1.012 (0.997–1.027)	1.011 (0.997–1.025)	1.004 (0.991–1.017)
Lag 02	1.006 (0.995–1.017)	1.004 (0.987–1.021)	1.007 (0.991–1.023)	0.994 (0.979–1.009)
Lag 03	1.002 (0.990–1.014)	1.001 (0.982–1.019)	1.007 (0.989–1.025)	0.989 (0.972–1.006)

* *p* < 0.05; AMI, acute myocardial infarction; CI, confidence interval. ^a^ Results adjusted for calendar time, daily mean temperature, dew-point temperature, relative humidity and day of week.

**Table 3:** Adjusted relative risk for daily cases of AMI event (95% CI) per 10 µg/m^3^ increase in PM2.5 at different lag days, stratified by quartiles of daily mean temperature levels at lag 0 ^ab^.

Temperature (°C)	Lag 0	Lag 1	Lag 2	Lag 3	Lag 01	Lag 02	Lag 03
<3.70	0.983 (0.964–1.002)	0.988 (0.972–1.005)	0.989 (0.973–1.005)	0.992 (0.977–1.008)	0.985 (0.965–1.004)	0.980 (0.960–1.001)	0.977 (0.956–0.999)
3.70–14.30	1.030 (1.014–1.046) *	1.006 (0.990–1.022)	1.002 (0.986–1.019)	1.000 (0.983–1.016)	1.024 (1.006–1.042) *	1.022 (1.002–1.042) *	1.018 (0.996–1.041)
14.30–22.40	1.017 (0.998–1.037)	1.007 (0.998–1.025)	1.011 (0.993–1.031)	1.002 (0.984–1.020)	1.015 (0.995–1.036)	1.019 (0.997–1.042)	1.018 (0.995–1.043)
≥22.40	1.003 (0.984–1.023)	1.011 (0.992–1.031)	1.000 (0.981–1.019)	0.999 (0.979–1.020)	1.008 (0.986–1.030)	0.999 (0.975–1.024)	0.994 (0.967–1.022)

* *p* < 0.05. ^a^ Results adjusted for calendar time, daily mean temperature, dew-point temperature, relative humidity and day of week. ^b^ Using the quartile of temperature as the cut-off value.

## Data Availability

Restrictions apply to the availability of these data.

## Supplementary Materials

The following are available online at https://www.mdpi.com/article/10.3390/ijerph18094822/s1, Table S1: Mean and the variance of daily AMI counts in our study, Table S2: Average air-pollutant concentration and weather conditions during the study period (2005 to 2014), Table S3: Newly diagnosed AMI patients in Korea from 2005 to 2014. Table S4: Adjustive relative risk for daily cases of AMI events (95% CI) per 1 °C increase in temperature, Table S5: Temperature level of Seoul in each month in our study, Table S6: Adjusted relative risk for daily cases of AMI event (95% CI) per 10 $\mu g/m^{3}$ increase in PM2.5 when temperature levels are between 3.70–14.30 °C, in single and co-pollutant models.
