# Supplementary material for: Effect of Short-Term Exposure to Fine Particulate Matter and Temperature on Acute Myocardial Infarction in Korea
Source: Int J Environ Res Public Health. 2021 Apr 30;18(9):4822. doi: 10.3390/ijerph18094822 (PMC8124364; doi:10.3390/ijerph18094822)
Supplement: Supplementary file 1 [file ijerph-18-04822-s001.zip › ijerph-1181692-supplementary.pdf]

**Supplementary Table S1.** Mean and the variance of daily AMI counts in our study.

| Scale | Value |
|---|---|
| Mean | 9.11 |
| Variance | 10.68 |

**Supplementary Table S2.** Average air-pollutant concentration and weather conditions during the study period (2005 to 2014).

| Value | All Seasons | Cold Season[b] | Warm Season[b] |
|---|---|---|---|
| PM2.5 [a] (**μg/m$^3$**) | 25.7 ± 14.2 | 27.7 ± 14.9 | 23.0 ± 12.5 |
| Meteorological indicators | | | |
| Daily average temperature (°C) | 12.7 ± 10.6 | 5.39 ± 7.55 | 22.8 ± 3.67 |
| Daily maximum temperature (°C) | 17.1 ± 10.8 | 9.83 ± 8.07 | 27.1 ± 3.81 |
| Daily minimum temperature (°C) | 8.96 ± 10.7 | 1.54 ± 7.32 | 19.2 ± 4.13 |
| Relative humidity (%) | 60.6 ± 15.0 | 55.7 ± 13.8 | 67.4 ± 13.9 |
| Dew point temperature (°C) | 4.55 ± 12.1 | −3.56 ± 8.70 | 15.8 ± 5.45 |

[a] PM2.5: particulate matter <2.5 μm in aerodynamic diameter. Summary statistics in Mean±SD. [b] $p$ < 0.001 for all comparisons between the cold and warm season.

**Supplementary Table S3.** Newly diagnosed AMI patients in Korea from 2005 to 2014.

| Value | 2005 | 2006 | 2007 | 2008 | 2009 | 2010 | 2011 | 2012 | 2013 | 2014 |
|---|---|---|---|---|---|---|---|---|---|---|
| AMI Patients | | | | | | | | | | |
| Overall | 17872 | 19852 | 19593 | 18428 | 18253 | 18054 | 18647 | 19526 | 20569 | 21773 |
| Seoul | 3598 | 3401 | 3345 | 3216 | 3217 | 3177 | 3173 | 3312 | 3345 | 3490 |
| Crude incidence [a] | | | | | | | | | | |
| Overall | 49.8 | 54.6 | 53.3 | 49.4 | 48.4 | 47.3 | 48.2 | 49.8 | 51.9 | 54.1 |
| Seoul | 46.4 | 43.4 | 42.4 | 40.4 | 40.0 | 39.5 | 39.3 | 40.9 | 41.2 | 42.7 |

[a] Incidence of newly diagnosed myocardial infarction are presented as 100,000 person-years.

**Supplementary Table S4.** Adjustive relative risk for daily cases of AMI events (95% CI) per 1 °C increase in temperature [a].

| Temperature Levels | Relative Risk (95% CI) |
|---|---|
| Mean temperature | 0.998 (0.978–1.017) |
| Minimum temperature | 0.999 (0.989–1.009) |
| Maximum temperature | 1.007 (0.997–1.016) |

[a] The model for this table is shown as follows: $\text{Log}[\text{E}(Y_t)] = \text{intercept} + \beta\text{Temperature} + \text{s}(\text{pm}) + \text{s}(\text{Calendar time}, 4 * 10) + \text{s}(\text{Dew point temperature}, 3) + \text{s}(\text{relative humidity}, 5)$ + day of week.

**Supplementary Table S5.** Temperature level of Seoul in each month in our study.

| Month | Daily Temperature (°C) | | |
|---|---|---|---|
| | **Mean** | **Maximum** | **Minimum** |
| January | −2.46 | 1.44 | −5.91 |
| February | 0.50 | 4.80 | −3.20 |
| March | 5.47 | 10.1 | −1.49 |
| April | 12.0 | 17.0 | 7.67 |
| May | 18.3 | 23.6 | 13.8 |
| June | 22.9 | 27.6 | 19.1 |
| July | 25.0 | 28.4 | 22.2 |
| August | 26.2 | 29.9 | 23.2 |
| September | 21.7 | 26.0 | 18.0 |
| October | 15.5 | 20.5 | 11.2 |
| November | 7.60 | 11.9 | 3.80 |
| December | −1.00 | 2.87 | −4.46 |

**Supplementary Table S6.** Adjusted relative risk for daily cases of AMI event (95% CI) per 10 μg/m$^3$ increase in PM2.5 when temperature levels are between 3.70-14.30°C**,** in single and co-pollutant models [ab.]

| Lag Day | RR (95% CI) | | | |
|---|---|---|---|---|
| | **None** | **+CO** | **+SO2** | **+NO2** |
| Lag 0 | 1.030 (1.014–1.046) * | 1.030 (1.007–1.053) * | 1.049 (1.025–1.074) * | 1.033 (1.012–1.054) * |
| Lag 1 | 1.006 (0.990–1.022) | 0.995 (0.972–1.018) | 1.002 (0.979–1.026) | 0.991 (0.970–1.011) |
| Lag 2 | 1.002 (0.986–1.019) | 0.992 (0.967–1.017) | 1.001 (0.976–1.026) | 0.989 (0.968–1.012) |
| Lag 3 | 1.000 (0.983–1.016) | 1.007 (0.982–1.032) | 1.006 (0.981–1.032) | 1.005 (0.983–1.027) |
| Lag 01 | 1.024 (1.006–1.042) * | 1.019 (0.992–1.046) | 1.033 (1.005–1.061) * | 1.015 (0.991–1.040) |
| Lag 02 | 1.022 (1.002–1.042) * | 1.012 (0.982–1.044) | 1.029 (0.997–1.061) | 1.005 (0.977–1.035) |
| Lag 03 | 1.018 (0.996–1.041) | 1.017 (0.984–1.051) | 1.030 (0.996–1.066) | 1.012 (0.980–1.045) |

* $p < 0.05$; AMI, acute myocardial infarction; CI, confidence interval. [a] Results adjusted for calendar time, daily mean temperature, dew-point temperature, relative humidity and day of week. [b] Using temperature levels at lag 0.
